# Echocardiographic Evaluation of Initial Ambrisentan Plus Phosphodiesterase Type 5 Inhibitor on Right Ventricular Pulmonary Artery Coupling in Severe Pulmonary Arterial Hypertension Patients

**DOI:** 10.3389/fcvm.2022.843606

**Published:** 2022-05-03

**Authors:** Wei-Fang Lan, Yan Deng, Bin Wei, Kai Huang, Ping Dai, Shan-Shan Xie, Dan-dan Wu

**Affiliations:** ^1^Department of Ultrasound, First Affiliated Hospital of Guangxi Medical University, Nanning, China; ^2^Department of Cardiology, First Affiliated Hospital of Guangxi Medical University, Nanning, China

**Keywords:** pulmonary arterial hypertension, right ventricular pulmonary arterial coupling, echocardiography, combination therapy, right ventricular remodeling

## Abstract

**Introduction:**

ambrisentan and phosphodiesterase type 5 inhibitor (PDE5i) have been approved for treating patients with pulmonary arterial hypertension (PAH). Echocardiographic right ventricular pulmonary artery coupling (RVPAC) has been shown to be a valid non-invasive and alternative measurement method to assess the predicted outcomes in PAH patients. The aim of this study was to study the effect and clinical correlates of initial ambrisentan plus PDE5i combination therapy on RVPAC in patients with severe PAH.

**Method and Results:**

We retrospectively studied and analyzed comprehensive clinical data, hemodynamics, and echocardiography in 27 patients with severe PAH before and after 6 months of initial combination therapy. Compared with the baseline, significant improvements in RVPAC ratios were observed, including RVFAC/PASP (0.31 ± 0.10 vs. 0.44 ± 0.15%/mmHg, *p* < 0.001), TAPSE/PASP (0.15 ± 0.05 vs. 0.21 ± 0.06 mm/mmHg, *p* = 0.001), S’/PASP (0.10 ± 0.03 vs. 0.14 ± 0.05 cm/s∙mmHg, *p* = 0.001), and RVSV/RVESV (0.79 ± 0.22 vs. 1.02 ± 0.20, *p* < 0.001). Functional status indices [World Health Organization functional classifications (WHO-FC) and 6 min walk distance (6MWD) and N-terminal pro B-type natriuretic peptide (NT-proBNP) levels] showed significant improvements. Right heart catheterization (RHC) evaluations for hemodynamic measurements between baseline and the 6–12 month follow-up were sPAP (96 ± 22 vs. 86 ± 24 mmHg, *p* = 0.002), mPAP (64 ± 18 vs. 56 ± 17 mmHg, *p* < 0.001) and TPVR (17.3 ± 6.7 vs. 12.1 ± 5.4 WU, *p* = 0.001). Simultaneously, significant associations between RVPAC ratios and NT-proBNP levels and WHO-FC and 6MWD were observed.

**Conclusion:**

Ambrisentan plus PDE-5i combination therapy resulted in a significant improvement in RVPAC in severe PAH. Importantly, RVPAC parameters correlated with known prognostic markers of PAH.

## Introduction

Pulmonary arterial hypertension (PAH) is a severe clinical syndrome characterized by increasing pulmonary vascular resistance resulting in chronic pressure overload of the right ventricle (RV) and ultimately leading to right heart failure and death (1, 2).

The treatment goal in PAH patients is to achieve good exercise capacity, reduce the RV afterload, keep the patient in WHO-FC II and maintain low mortality rates whenever possible (3). Current research studies on potential therapeutic targets and the development of new PAH-targeted therapies focused on the endothelium, nitric oxide, and prostacyclin pathways have contributed to improved survival in patients with PAH (2, 3). Ambrisentan is an endothelin-specific receptor antagonist (ERA) approved for the treatment of PAH. Phosphodiesterase type 5 inhibitor (PDE-5i), such as sildenafil, are available therapies to influence the nitric oxide pathway. Initial or early combination therapy (generally ambrisentan and PDE-5i) is an attractive option and is currently recommended for treating patients with PAH, especially severe PAH (3, 4).

The main prognostic factor of symptoms and outcome in PAH is related to RV function (3, 5). However, merely assessing RV function can provide limited insight into RV adaptation and chronic volume overload and does not take into account the effects of pulmonary circulation (6). Right ventricular pulmonary arterial coupling (RVPAC) based on the matching of RV function and its vascular circulation can early and precisely reflect the status of overloaded RV function (6, 7). The gold standard of assessing RVPAC is the ratio of end-systolic elastance to pulmonary arterial elastances (Ees/Ea), which has been evaluated by invasive right heart catheterization (RHC). Ideally, coupling is maintained at resting conditions in patients with adaptive RV. Only in the late stages of pressure overload does uncoupling occur (6, 8).

Echocardiography is a non-invasive ultrasound technique and provides RV function parameters such as tricuspid annular plane systolic excursion (TAPSE), right ventricular fractional area change (RVFAC), tricuspid annular peak systolic tissue Doppler velocity (TASV or S’) and pulmonary artery systolic pressure (PASP) for estimating hemodynamic characteristics or improving RV function in patients with PAH (3, 9). Recently, some studies demonstrated that the ratios of TAPSE to pulmonary artery systolic pressure (PASP) were closely related to prognostic markers and have been considered a surrogate of Ees/Ea (10–14). The TASV/RVSP ratio, as a determinant of poor prognosis, is associated with short-term and long-term mortality (15). In addition, another ratio of RV stroke volume (RVSV) to RV end-systolic volume (RVESV), measured by 3-dimensional echocardiography (3-DE), has the same capacity and is a significant predictor of adverse clinical events for pediatric patients with PAH (16). However, data concerning the evaluation of right ventricular function and RVPAC in severe PAH patients after ambrisentan and PDE-5i combination therapy are scarce.

Our study aims to (1) determine changes in RV function and RVPAC in response to combination therapy with initial ambrisentan plus PDE-5i in patients with severe PAH and (2) evaluate the relationship between RVPAC and clinical parameters.

## Materials and Methods

### Study Population

A retrospective study was conducted in treatment-naive patients and their medical records from June 2019 to February 2022 were collected and reviewed at the First Affiliated Hospital of Guangxi Medical University. The patients’ diagnoses were confirmed by RHC, and confirmed by criteria such as a mean pulmonary artery pressure (mPAP) > 25 mmHg, a pulmonary vascular resistance (PVR) > 5 Wood Units (WU), and pulmonary arterial wedge pressure (PAWP) or left ventricular end diastolic pressure of < 15 mmHg (3). Based on relevant literature, at least two experts confirmed, through a comprehensive data review of severe PAH-related assessments, severe PAH in patients with reduced RV function as assessed by echocardiography and right heart catheterization (higher right atrial pressure, lower cardiac index, increased pulmonary vascular resistance index), a WHO FC III or IV, and a significantly elevated N-terminal-Pro-Brain-Natriuretic Peptide (NT-proBNP) level (3, 4, 17). Disease progression and the presence or absence of signs of right heart failure were also considered. They were treated with an upfront combination of ambrisentan and PDE-5i.

The following were exclusion criteria: (1) total lung capacity of pulmonary function test was lower than the 60% estimated value; (2) suffering from interstitial lung disease, pulmonary hypertension due to lung diseases or chronic thromboembolic disease; (3) pulmonary hypertension due to left heart disease, including left ventricular systolic or diastolic dysfunction, valvular disease (moderate to severe stenosis or insufficiency confirmed by echocardiography), cardiomyopathies; (4) pulmonary valve or pulmonary arterial stenosis; and (5) autonomic hypotension, severe hepatic and renal impairment.

Patients who fulfilled the above criteria and had PAH confirmed by RHC but who had not yet received PAH-specific therapy were enrolled.

### Clinical Data

At the baseline, patient demographics, clinical status and treatment strategies were collected by review of electronic medical records. Heart function was assessed by World Health Organization functional classifications (WHO-FC). A 6 min walk distance (6MWD) was performed according to American Thoracic Society guidelines (18).

Biochemical markers were obtained, and the level of plasma N-terminal pro B-type natriuretic peptide (NT-proBNP) was measured by an enzyme-linked immunosorbent assay using a COBAS 6000 E601 immunoassay analyzer and an Elecsys proBNP II reagent kit (Roche Diagnostics GmbH, Mannheim, Germany).

### Right Heart Catheterization

Right heart catheterization was performed using a balloon wedge catheter through the femoral vein or internal jugular vein by standard methods at the first diagnosis of PAH (19). At the follow-up, RHC was performed using MPA2 angiographic catheter (6F, Cordis Corporation, Miami Lakes, FL, United States) under fluoroscopic guidance. Hemodynamic measurements obtained by RHC included mean pulmonary arterial pressure (mPAP), systolic pulmonary arterial pressure (sPAP), diastolic pulmonary arterial pressure (dPAP), mean right ventricular pressure (mRVP), systolic right ventricular pressure (sRVP), diastolic right ventricular pressure (dRVP), mean right atrial pressure (mRAP), heart rate and pulmonary arterial wedge pressure (PAWP). Cardiac output (CO) was divided by heart rate to obtain stroke volume. The cardiac index (CI) was divided by body surface area (BSA) to CO. Total pulmonary vascular resistance (TPVR) was divided by CO to mPAP. Pulmonary vascular resistance (PVR) was calculated as (mPAP − PAWP)/CO.

### Transthoracic Echocardiography

Transthoracic echocardiography was performed on all patients with an available echocardiographic Philips EPIC 7C (Philips Healthcare, Andover, MA, United States), and 3DE RV data were digitally analyzed using an advanced data quality quantification system (Philips Healthcare, Andover, MA, United States). All parameters were performed both at baseline and after 6 months of combination therapy in patients with severe PAH according to the recommendations for echocardiographic assessment of the right heart by the American Society of Echocardiography chamber guidelines (9, 20).

TAPSE was defined as the difference between the displacement of the RV lateral annulus from end-diastole to end-systole obtained by M-mode imaging in the apical four-chamber view centered on the RV. S’ was defined as tricuspid annular systolic velocity derived from tissue Doppler transthoracic echocardiography. PASP was calculated using the maximal tricuspid regurgitation velocity obtained from continuous wave Doppler and integrated into the modified Bernoulli equation [PASP = 4 (TRV)^2^ + RAP]. Right atrial pressure was estimated through the inferior vena cava (IVC) size and variability with respiration. RAP is equal to 3 mmHg when the IVC size ≤ 2.1 cm and variability with respiration (> 50% diameter change with inspiration), equal to 8 mmHg when the IVC size > 2.1 cm and variability with respiration (> 50% diameter change with inspiration), and equal to 15 mmHg when the IVC size > 2.1 cm and variability with respiration (< 50% diameter change with inspiration), according to the guidelines (20).

The 3D data were transferred and analyzed using an advanced data quality quantification system (QLAB version 12.0; Philips Healthcare) to automatically generate RV volumes and functional indices. Specifically, there were acquired in 3 steps. First, the RV basal short-axis and apical two- and four-chamber views were extracted by an advanced data quality quantification system equipped with 3D viewer software, and the software initially identified RV long-axis landmarks in end-diastole in the apical two- and four-chamber views. Next, the tricuspid valve annulus hinge points and the RV endocardial surfaces were automatically defined and tracked throughout the cardiac cycle (inclusion of papillary muscles and trabeculae in the cavity volume). The automatic tracings were manually adjusted if correction was needed. Finally, the RV volume–time curve was automatically constructed, and RV volumes and functional indices were obtained, including 3D RV end-diastolic volume (RVEDV), RV end-systolic volume (RVESV), RV stroke volume (RVSV), and RV ejection fraction (RVEF) was calculated using the standard formula: RVEF = (RVEDV-RVESV)/RVEDV (20). RV fractional area change (RVFAC) was defined as the percentage of the RV area from end-diastolic to end-systolic obtained by 3DE in the apical four-chamber view centered on the RV.

These measurements were performed at baseline and after 6 to 12 months of combination therapy.

### Variability Analysis of 3DE Right Ventricle Measurements

Intraobserver and interobserver variability of the newer measures of RV volume and function assessment were evaluated in 10 randomly selected patients by 2 different investigators. For variability analysis, similar views were used but not necessarily the same beat, each blinded to the results obtained by the other. For intraobserver variability, RV measurements were repeated by the same investigator 6 months later. Reproducibility of the RV measurements was assessed using intraclass correlation coefficients. An intraclass correlation coefficient > 0.9 was considered to represent a high degree of reliability of the strain measurements.

### Assessment of Right Ventricular Function and Right Ventricular Pulmonary Artery Coupling

RV systolic function was evaluated using RVFAC, S’, TAPSE and RVEF. RV diastolic function was evaluated by the Doppler velocity of the transtricuspid flow (E), lateral tricuspid annular diastolic tissue Doppler velocity (E’) and tricuspid annular E/E’ ratio. The TAPSE/PASP, RVFAC/PASP, RVSV/RVESV and S’/PASP ratios were considered indices of RVPAC (10–16).

### Statistical Analysis

Statistical analyses were performed utilizing SPSS statistical software (version 26, IBM Corporation, Armonk, NY, United States). Normal and non-normal distributions were determined using the Shapiro–Wilk test and visual analysis (P-P plots, Q-Q plots). Continuous variables are presented as the mean ± standard deviation or median and interquartile range (IQR; 25th–75th percentiles), and categorical variables are expressed as numbers and percentages. NT-proBNP values were log-transformed for analyses. The paired Student’s *t*-test, chi-square test, and Wilcoxon signed rank tests were used in patients for measurements at 2 time points. RV volumes adjusted for body surface area (BSA). Correlations between RVPAC indices and NT-proBNP, 6MWD, WHO FC were evaluated by the Spearman rank correlation coefficient or Pearson correlation coefficient. For all tests, a *p* value less than 0.05 was considered statistically significant.

## Results

### Baseline Demographic and Clinical Characteristics

We retrospectively reviewed 27 patients (median age 37 years, 85% female) who were provided with comprehensive data in this study, including idiopathic PAH (*n* = 15, 55%), congenital heart disease-associated PAH (*n* = 8, 30%) and systemic lupus erythematosus-associated PAH (*n* = 4, 15%). During the follow-up period, 21 patients were treated with ambrisentan plus tadalafil, and 6 patients were treated with ambrisentan plus sildenafil. In patients who had WHO FC IV at baseline, there were 4 patients treated with ambrisentan and PDE5i for intermediate risk (3), 2 patients for high risk were inoperable with initial combination therapy including i.v. prostacyclin because of the expensive price. The demographics and baseline characteristics of the study patients are shown in Table 1.

**TABLE 1:** Baseline demographics and clinical characteristics.

Total cohort	
Patients, n	27
Age, y	37 ± 14
Sex (female), %	23(85)
BSA, m^2^	1.49 ± 0.19
SBP, mmHg	108 ± 14
DBP, mmHg	72 ± 10
HR, bpm	90 ± 12
**Clinical presentation, n**	
Exercise-induced symptoms	26 (96)
Syncope	3 (11)
Peripheral edema	9 (33)
Chronic cough	10 (37)
Hemoptysis	1 (4)
**Clinical signs, n**	
Enlargement of the heart	16 (59)
Accentuation of the P2	25 (93)
Right heart failure signs	12 (44)
Cardiac murmur	19 (70)
Cyanopathy	5 (19)
**Electrocardiogram, n**	
Right ventricular hypertrophy	12 (44)
Right bundle branch block	8 (30)
Arrhythmia	4 (15)
ST -T change	11 (41)
Normal	8 (30)
LVEF, %	71 ± 8

*BSA, body surface area; SBP, systolic blood pressure; DBP, diastolic blood pressure; HR, heart rate; P2, pulmonary component of the second heart sound; LVEF, left ventricular ejection fraction.*

### Clinical Status Change

After 6 months of combination therapy, the majority of patients improved their WHO FC, and 20 patients (74%) reached WHO-FC I or II (Table 2). NT-proBNP levels were significantly decreased from 1,690.0 (510.3–3,568.0) to 112.0 (1 9.85–1,936.0) pg/mL (*p* < 0.001). Simultaneously, a statistically significant difference was observed in the 6MWD (baseline 323 ± 125 vs. 391 ± 115 m after treatment, *p* = 0.004) (Table 2).

**TABLE 2:** Functional status, hemodynamic at baseline and follow-up.

Variables	Baseline	Follow-up	*P* value
**Functional status (*n* = 27)**			
WHO FC I/II/III/IV, n	4/6/11/6	7/13/5/2	0.002
6MWD, m	323 ± 125	391 ± 115	0.004
NT-proBNP, pg/mL	1690.0 (510.3–3,568.0)	112.0 (19.85–1,936.0)	<0.001
**Right heart catheterization (*n* = 23)**			
mPAP, mmHg	64 ± 18	56 ± 17	<0.001
sPAP, mmHg	96 ± 22	86 ± 24	0.002
mRAP, mmHg	9 ± 4	9 ± 5	0.948
TPVR, WU	17.3 ± 6.7	12.1 ± 5.4	0.001
PAWP, mmHg	8 ± 2	–	–
PVR, WU	13 ± 6	–	–
CI, L/min/m^2^	2.5 (2.0–3.5)	3.0 (2.5–4.0)	0.110

*WHO-FC, World Health Organization functional class; 6MWD, 6 min walk distance; NT-proBNP, N-terminal pro B-type natriuretic peptide; sPAP, systolic pulmonary artery pressure; mPAP, mean pulmonary artery pressure; mRAP, mean right atrial pressure; PAWP, pulmonary arterial wedge pressure; (T) PVR, (total) pulmonary vascular resistance; CI, cardiac index.*

### Hemodynamic Improvement

**FIGURE 1:**
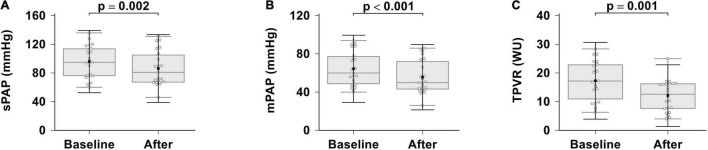
Effects of ambrisentan plus PDE-5i combination therapy on hemodynamics. **(A)** sPAP; **(B)** mPAP; **(C)** TPVR. sPAP, systolic pulmonary artery pressure; mPAP, mean pulmonary artery pressure; TPVR, total pulmonary vascular resistance.

The effects of combination therapy on hemodynamics are presented in Table 2 and Figure 1. Patients showed significant hemodynamics improvements after 6 months of initial combination therapy. Significant improvements in mPAP, sPAP and TPVR were observed (*p* < 0.001, *p* = 0.002, *p* = 0.001, respectively). We did not observe any significant changes in CI (*p* = 0.110). We observed a baseline median cardiac index of 2.5 L/min/m^2^, which was indicative of RV dysfunction. At the same time, we did not observe any significant changes in mRAP (baseline 9 ± 3 mmHg vs. 8 ± 5 mmHg after treatment, *p* = 0.948).

### Right Ventricular Function and Pulmonary Artery Systolic Pressure Improvement

**FIGURE 2:**
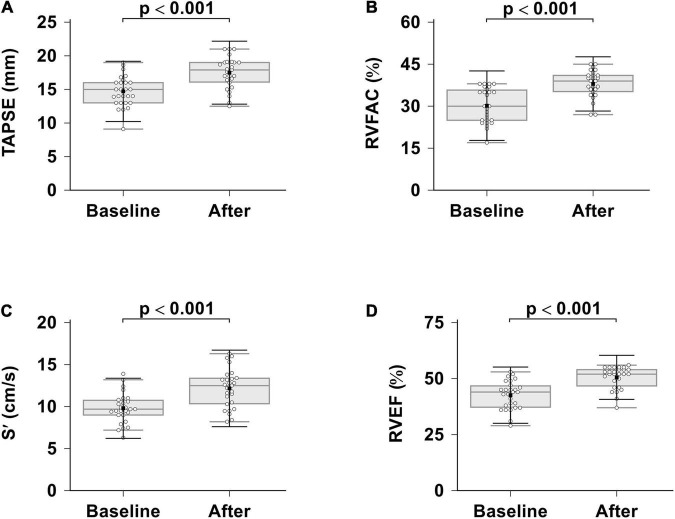
Effects of ambrisentan plus PDE-5i combination therapy on RV systolic function. **(A)** TAPSE; **(B)** RVFAC; **(C)** S’; **(D)** RVEF. TAPSE, tricuspid annular plane systolic excursion; RVFAC, right ventricular fractional area change; S’, tricuspid annular systolic velocity; RVEF, right ventricular ejection fraction.

Routine RV echocardiographic parameters differenced with respect to ambrisentan plus PDE-5i combination therapy are summarized in Table 3 and Figure 2. RV systolic function was significantly improved in patients with severe PAH compared to the baseline, as assessed by S’ (*p* < 0.001), TAPSE (*p* < 0.001), RVEF (*p* < 0.001), and RVFAC (*p* < 0.001).

**TABLE 3:** Echocardiographic parameters at baseline and follow-up.

Variables (*n* = 27)	Baseline	Follow-up	*P* value
RV wall thickness, mm	8 (6–8)	7 (6–8)	0.713
S’, cm/s	9.8 ± 1.8	12.2 ± 2.3	<0.001
E, cm/s	55.0 (42.0–80.0)	57.0 (42.0–77.0)	0.770
E’, cm/s	9.4 (6.9–12.3)	9.0 (7.1–14.6)	0.259
E/E’ ratio	7.1 ± 2.6	6.8 ± 3.0	0.664
TAPSE, mm	14.6 ± 2.1	17.5 ± 2.4	<0.001
TRV, cm/s	4.8 ± 0.7	4.4 ± 0.7	0.021
Tricuspid regurgitation, n (mild, moderate, severe)	12/9/6	15/8/4	0.166
PASP, mmHg	103 ± 29	90 ± 25	0.022
RVSV, mL	47 ± 18	49 ± 17	0.523
RVESV, mL	63 ± 24	51 ± 23	0.010
RVEDV, mL	110 ± 39	98 ± 38	0.076
RVSVi, mL/m^2^	32 ± 2	33 ± 2	0.622
RVESVi, mL/m^2^	42 ± 16	34 ± 14	0.004
RVEDVi, mL/m^2^	74 ± 5	66 ± 4	0.041
RVCO, L/min	4.1 ± 1.8	4.5 ± 2.0	0.232
RVEF, %	43 ± 6	51 ± 5	<0.001
RVFAC, %	30 ± 6	37 ± 6	<0.001

*S’, tricuspid annular systolic velocity; E/e, tricuspid early diastolic transmitral flow velocity to averaged annular early diastolic velocity ratio; TAPSE, tricuspid annular plane systolic excursion; TRV, tricuspid regurgitation velocity; PASP, systolic pulmonary artery pressure; RVEDV, right ventricular end-diastolic volume; RVESV, right ventricular end-systolic volume; RVSV, right ventricular stroke volume; RVEDVi, right ventricular end-diastolic volume indexed; RVESVi, right ventricular end-systolic volume indexed; RVSVi, right ventricular stroke volume indexed; RVCO, right ventricular cardiac output; RVEF, right ventricular ejection fraction; RVFAC, right ventricular fractional area change.*

RV diastolic function that assessed by the tricuspid annular E/E’ ratio was not different compared to the baseline (baseline 7.1 ± 2.6 vs. 6.8 ± 3.0 after treatment, *p* = 0.664) and remained impaired. There was a trend that showed a decrease in RVEDV, whereas RVSV was unchanged after treatment; RVESV significantly decreased. After adjusting for BSA, RVEDVi significantly decreased. At the same time, an RV echocardiography performed at 6 months revealed slightly significant reduction in TRV (*p* = 0.021) and estimated PASP (*p* = 0.022) but not in tricuspid regurgitation (*p* = 0.166).

### Right Ventricular Pulmonary Artery Coupling Improvement

**FIGURE 3:**
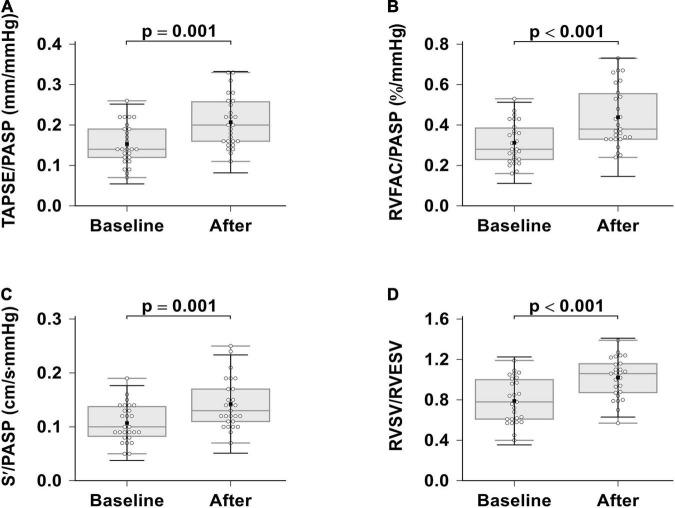
Effects of ambrisentan plus PDE-5i combination therapy on right ventricular pulmonary arterial coupling. **(A)** TAPSE/PASP; **(B)** RVFAC/PASP; **(C)** S’/PASP; **(D)** RVSV/RVESV. TAPSE, tricuspid annular plane systolic excursion; RVFAC, right ventricular fractional area change, S’, tricuspid annular systolic velocity; PASP, systolic pulmonary artery pressure, RVEF, right ventricular ejection fraction; RVESV, right ventricular end-systolic volume; RVSV, right ventricular stroke volume.

In this study, we looked for RVPAC changes induced by ambrisentan plus PDE-5i combination therapy (Table 4 and Figure 3). All RVPAC ratios (RVFAC/PASP, *p* < 0.001; RVSV/RVESV, *p* < 0.001; TAPSE/PASP, *p* = 0.001; S’/PASP, *p* = 0.001) were significantly increased. This indicates that combination therapy may result in significant improvements in RV function and decreases in RVPAC for patients with severe PAH.

**TABLE 4:** Right ventricular pulmonary arterial coupling at baseline and follow-up.

RVPAC	Baseline	Follow-up	*P* value
RVFAC/PASP, %/mmHg	0.31 ± 0.10	0.44 ± 0.15	<0.001
TAPSE/PASP, mm/mmHg	0.15 ± 0.05	0.21 ± 0.06	0.001
S’/PASP, cm/s⋅mmHg	0.10 ± 0.03	0.14 ± 0.05	0.001
RVSV/RVESV	0.79 ± 0.22	1.02 ± 0.20	<0.001

*RVPAC, Right ventricular pulmonary arterial coupling; S’, tricuspid annular systolic velocity; TAPSE, tricuspid annular plane systolic excursion; PASP, systolic pulmonary artery pressure; RVESV, right ventricular end-systolic volume; RVSV, right ventricular stroke volume; RVFAC, right ventricular fractional area change.*

### Relationship Between Clinical Parameters and Right Ventricular Pulmonary Artery Coupling

**FIGURE 4:**
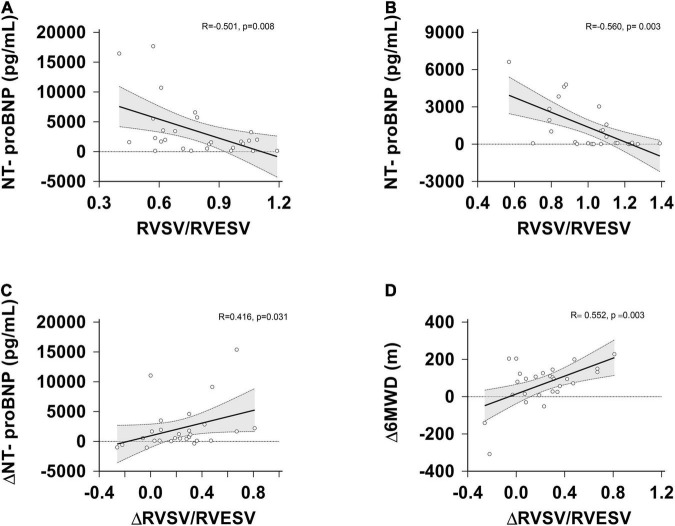
Correlation between RVSV/RVESV, △RVSV/RVESV, NT-proBNP, △NT-proBNP and △6MWD at the baseline and follow-up assessment. **(A)** RVSV/RVESV vs. NT-proBNP levels at the baseline; **(B)** RVSV/RVESV vs. NT-proBNP levels at the follow-up; **(C)** changes in RVSV/RVESV vs. changes in NT-proBNP levels. **(D)** changes in RVSV/RVESV vs. changes in the 6MWD. △, change; RVESV, right ventricular end-systolic volume; RVSV, right ventricular stroke volume; NT-proBNP, N-terminal pro B-type natriuretic peptide.

The correlations between 6MWD, NT-proBNP levels, WHO-FC and RVPAC parameters were showed in Table 5 and Figure 4. At the baseline, NT-proBNP levels had a significantly negative correlation with the SV/ESV and RVFAC/PASP ratio (*p* = 0.008, *p* = 0.037, respectively). The rest of measures had no significant correlation with the baseline functional parameters.

**TABLE 5:** Correlation of right ventricular pulmonary arterial coupling to functional status.

RVPAC	Correlation with NT-proBNP		Correlation with WHO FC		Correlation with 6MWD	
	R	*P* value	R	*P* value	R	*P* value
**Baseline RVPAC vs. baseline functional status**						
TAPSE/PASP	−0.274	0.167	−0.010	0.962	0.210	0.294
RVFAC/PASP	−0.403	0.037	−0.020	0.923	0.207	0.300
S’/PASP	−0.231	0.247	−0.007	0.965	0.202	0.312
RVSV/RVESV	−0.501	0.008	−0.181	0.366	0.270	0.173
**Follow-up RVPAC vs. follow-up functional status**						
TAPSE/PASP	−0.129	0.521	−0.399	0.039	0.468	0.013
RVFAC/PASP	−0.177	0.377	−0.417	0.031	0.482	0.011
S’/PASP	−0.310	0.116	−0.425	0.027	0.474	0.012
RVSV/RVESV	−0.560	0.003	−0.020	0.920	0.236	0.235
**Changes in RVPAC vs. changes in functional status**						
△TAPSE/PASP	0.446	0.020	0.015	0.940	0.445	0.020
△RVFAC/PASP	0.359	0.066	0.076	0.706	0.306	0.117
△S’/PASP	0.430	0.025	0.240	0.906	0.431	0.025
△RVSV/RVESV	0.416	0.031	0.046	0.818	0.552	0.003

*Correlation between right ventricular pulmonary arterial coupling ratios and functional status was evaluated by the Spearman rank correlation coefficient or Pearson correlation coefficient.*

*△, change; 6MWD, 6 min walk distance; WHO-FC, World Health Organization functional class; NT-proBNP, N-terminal pro B-type natriuretic peptide. RVPAC, Right ventricular pulmonary arterial coupling; S’, tricuspid annular systolic velocity; TAPSE, tricuspid annular plane systolic excursion; PASP, systolic pulmonary artery pressure; RVESV, right ventricular end-systolic volume; RVSV, right ventricular stroke volume; RVFAC, right ventricular fractional area change.*

After treatment, NT-proBNP levels still had a significantly negative correlation with the SV/ESV ratio (*r* = −0.560; *p* = 0.003), but had no significant correlation with the TAPSE/PASP, RVFAC/PASP, S’/PASP ratios. WHO-FC was significantly negatively correlated with the TAPSE/PASP, RVFAC/PASP, S’/PASP ratios (*p* = 0.039, *p* = 0.031 and *p* = 0.027, respectively), but had no significant correlation with SV/ESV ratio. The 6MWD was significantly positively correlated with the TAPSE/PASP, RVFAC/PASP, S’/PASP ratio (*p* = 0.013, *p* = 0.011 and *p* = 0.012, respectively), but had no significant correlation with the SV/ESV ratio.

Interestingly, the improvement in TAPSE/PASP, S’/PASP, RVSV/RVESV correlated with a reduction in NT-proBNP levels and an increase in the 6MWD. But there is not any relationship between the baseline coupling and follow-up functional state.

### Reproducibility

The results for the intraobserver and interobserver variability for 3DE RV volume and function assessment upon repeated measurements in 10 random patients were shown in Table 6. ICC values were higher for intraobserver and interobserver variability when the same images were analyzed by the two different cardiologists.

**TABLE 6:** ICCs for intra- and interobserver variability for 3DE RV measurements.

Variable	Interobserver variability		Intraobserver variability	
	ICC	95% CI	ICC	95% CI
RVEDV	0.990	0.960–0.997	0.991	0.965–0.998
RVESV	0.975	0.909–0.998	0.993	0.972–0.998
RVSV	0.991	0.910–0.994	0.972	0.888–0.993
RVEF	0.935	0.746–0.984	0.949	0.802–0.987
RVCO	0.988	0.952–0.997	0.987	0.947–0.997

*ICC, intraclass correlation coefficient; RVEDV, right ventricular end-diastolic volume; RVESV, right ventricular end-systolic volume; RVSV, right ventricular stroke volume; RVCO, right ventricular cardiac output; RVEF, right ventricular ejection fraction.*

## Discussion

In the present study, a significant improvement in hemodynamics, RV function and RVPAC was demonstrated as a result of 6 months of initial ambrisentan plus PDE-5i combination therapy. We also demonstrated that RVPAC parameters correlated with various clinical parameters such as the 6MWD, NT-proBNP levels and WHO-FC.

Current therapeutic options targeting prostacyclin, nitric oxide, or the endothelin pathway are involved in PAH patients. With regard to the updated treatment for PAH, the latest guidelines give a strong recommendation for the use of combination therapy, even for patients with milder WHO-FC II–III PAH (3). Previous reports offered important evidence and manifested a statistically significant greater decrease in hemodynamics and an enhancement in functional overall survival rates after initial combination therapy (21–25). Several studies showed the effect of combination therapy on afterload and RV reverse remodeling in PAH (25–29). Similar as previous studies, we observed a prominent elevation in RV function, including the S’, RVEF, RVFAC, TAPSE and RVPAC ratios, a reduction in pulmonary artery pressure and RV afterload after combination therapy. In addition, 6 patients had WHO FC IV at baseline, and the current guidelines emphasize the initiation of prostacyclin analog therapy for these patients. The initial combination therapy strategy based on the comprehensive risk assessment in guidelines for the diagnosis and treatment of pulmonary hypertension (3). The patients can be classified as low risk, intermediate risk or high risk for clinical worsening or death. There were 4 patients treated with ambrisentan and PDE5i for intermediate risk, 2 patients for high risk were inoperable with initial triple combination therapy including i.v. treprostinil. D’Alto et al. showed that triple upfront combination therapy with ambrisentan, tadalafil, and subcutaneous treprostinil in severe non-reversible PAH is associated with considerable clinical and hemodynamic improvement and RV reverse remodeling.

Compared with the baseline, our study showed differences in WHO-FC and 6MWD after 6–12 months of initial combination therapy. This finding is consistent with the results of the AMBITION trial in which significant differences were observed in favor of combination therapy (21, 24). Furthermore, the differences in CI and mRAP were not significant. In our study, patients had a baseline median CI of 2.5 L/min/m^2^, undifferentiated mRAP and a median follow-up E/E’ of 6.8, which was indicative of RV dysfunction (3, 9). The reason CI was not improved may be that there was a subset of patients with severe tricuspid regurgitation. Our results did not show an improvement in tricuspid regurgitation. A previous report showed that tricuspid regurgitation progression was associated with worsening pulmonary hypertension and adverse RV and TV apparatus remodel in patients with PAH (30).

Regarding cardiac volumes, we observed decreases in RVEDVi and RVESVi, while RVSVi remained unchanged after treatment. These findings are partly similar to those found in previous research (26), Van de Veerdonk et al. showed that RV volumes (RVSV, RVESV, RVEDV) improved after combination therapy compared with the monotherapy group. Although reverse remodeling of the right heart is associated with functional improvement, moderate improvements in RV function may not result in a decrease in right heart size (31). We hypothesize that the consequence in this study may be due to the RV maladaptive remodeling that occurred in our severe PAH patients compared to those in an earlier stage of PAH. Usually, RV remodeling in PAH differentiates 2 patterns of ventricular remodeling: adaptive and maladaptive remodeling (32). Adaptive remodeling resembles the fetal RV phenotype and is characterized by concentric hypertrophy with slight eccentric dilatation and fibrosis, preserved systolic and diastolic function, and maintained of normal cardiac output and filling pressures (33–35). In these subjects, the RV is more capable of coping with increased RV afterload and is characterized by concentric remodeling, probably because a fetal phenotype is maintained throughout life, therefore developing compensatory hypertrophy over dilatation. Conversely, maladaptive remodeling causes eccentric dilatation, excessive fibrosis and worse systolic and diastolic function, in addition to reducing the ejection fraction, cardiac output and elevating filling pressures (32, 34). A previous report showed that the reversal of RV remodeling in IPAH patients after 1-year of targeted treatment, is difficult and complex (36). We speculate that a potential cause for the lack of improvement in RVSV is most likely related to the relatively small number of patients in our study.

Previous research is limited to estimating routine RV echocardiographic parameters or invasive measurements. No published studies have examined the effects of echocardiographic measures of RVPAC after initial ambrisentan plus PDE-5i combination therapy in severe PAH patients.

The gold-standard method to assess RVPAC in clinical practice should use RHC and was used to assess treatment response in the ERS/ESC guidelines (3). Not all medical institutions set up routine hemodynamic evaluation centers. A number of studies indicate that non-invasive echocardiography may be superior to invasive RHC or relatively expensive cardiac magnetic resonance imaging (CMRI) for routine follow-up of patients with PAH.

In our study, the TAPSE/PASP, RVFAC/PASP, RVSV/RVESV and S’/PASP ratios were considered indices of RVPAC. Some previous studies showed that TAPSE/PASP were crucial surrogates for describing RVPAC and emerged as an independent predictor of Ees/Ea (10, 11, 37). A correlation analysis revealed a significant direct relationship between the TAPSE/PASP ratio and pro-BNP levels. While the TAPSE/PASP ratio has been substantiated in patients with PAH, only a few studies have explored the significance of the other parameters. Several other ratios have been proposed to estimate RVPAC. The FAC/RV systolic pressure (RVSP) or TAPSE/RVSP ratio has been shown to be of superior prognostic value compared with RV systolic function (FAC or TAPSE) in other studies (38–40). Jentzer et al. (15) described the relationship between the lower tricuspid annular systolic velocity (TASV)/RVSP ratio derived from Doppler transthoracic echocardiography and short-term and long-term mortality in cardiac intensive care unit patients. Similarly, because Ees and Ea share a common pressure term, coupling can be simplified as SV/ESV and is quantifiable with magnetic resonance (41). A cutoff value of approximately 0.54 for SV/ESV prediction has been validated as a predictor of poor outcomes in patients with PH (16). The SV/ESV ratio as a volume estimate of coupling ratio correlates with RV strain and is a strong predictor of adverse clinical events in pediatric PH (33). The SV/ESV ratio was suitable for patients with limited TR signals.

In this study, the mean baseline TAPSE/PASP was fairly low at 0.14 mm/mmHg and had a lower mean baseline RVSV of 47 mL, which suggested a rather sick PAH patient population. However, baseline 3D RV volumes do not seem to be consistent with such a low TAPSE/PASP. According to Telio et al. (37), the patient population had severe PH with a mean Ees/Ea ratio of 0.70, a mean TAPSE/PASP ratio of 0.28 mm/mmHg (0.19–0.42), a TAPSE/PASP cutoff of 0.31 mm/mmHg, an RV end-diastolic volume/body surface area of 111 mL/m^2^ (88–144), and RVSV of 71 mL (59–95), which implied that they were already in an RV-PA uncoupling state. Compared with Gerges et al. (42) who examined TAPSE/PASP in patients with combined pre- and postcapillary PH and idiopathic PAH, the mean TAPSE/PASP ratio in that study of patients with idiopathic PAH was 0.14 ± 0.11 mm/mmHg. Whether these results can be extrapolated for patients with other etiologies of PH, as well for different stages of severity of PH, the conclusion is guarded. One possible explanation for this result is that RV volumes tend to reach a minimum as RV progresses. dysfunction. Another possibility that cannot be ruled out is that we overestimated the PASP that was calculated using the modified Bernoulli equation [PASP = 4 (TRV)^2^ + RAP].

In addition, NT-proBNP, WHO-FC and 6MWD seem to be stronger predictors of prognosis and survival (3). We found that the SV/ESV ratio had a stronger negative correlation with log-transformed NT-proBNP levels. The ratios of RVFAC/PASP, TAPSE/PASP and S’/PASP were correlated with WHO-FC and 6MWD. In contrast, our results failed to demonstrate a correlation between the TAPSE/PASP ratio and NT-proBNP levels. However, we remain firmly convinced of the importance of the TAPSE/PASP ratio in RV function and clinical outcome. Routine measurements are limited because of the complex shape of the RV and the interaction between the RV and pulmonary vascular system. While multiple non-invasive parameters to approximate RV function have been studied, one of the more promising methods is the use of the TAPSE/PASP ratio (10). A better RVPAC might reflect both a significant reversible remodeling of the RV and a fixed decrease in pulmonary vascular resistances. Clinically, these patients probably have reversible structural damage and are more prone to benefit from ambrisentan plus PDE-5i combination therapy. The TAPSE/PASP ratio has been validated in patients with PAH; however, the other parameters have not and there are minimal data on many of them. The means by which to interpret these other parameters are unclear, and their relevance is questionable. Non-invasive imaging of RV structure and function is essential to monitoring patients with PAH and appraising the RV response to pulmonary vascular remodeling. Our study attempts to provide potential useful parameters of RV-PA coupling for characterizing RV function and clinical improvement from a new perspective.

No prior study has reported on the effects of ambrisentan plus PDE-5i combination therapy on RV remodeling and RVPAC in severe PAH patients by non-invasive echocardiography, which provides important information about the effectiveness of combination therapy. Our study indicate that ambrisentan plus PDE-5i combination therapy may result in significant improvements in RV systolic function and RVPAC for severe PAH patients. Most importantly, RVPAC parameters were related to the 6MWD, NT-proBNP levels and WHO FC, which established prognostic markers in PAH.

### Strengths and Limitations

The advantage of the current study lies in detailed information, including clinical functional status, laboratory data, and invasive hemodynamic and non-invasive echocardiography data. There are several limitations to this study. First, the present study was performed as a retrospective observational, single-center cohort, small population study. Due to the small sample size, we were unable to perform quantitative analysis and identify risk factors. Second, considering the high cost of, balloon wedge catheter, pulmonary arterial wedge pressure (PAWP) during RHC was only performed at the first diagnosis (i.e., baseline) in our study. At follow-up measurements, RHC was performed using more economical MPA2 angiographic catheter under fluoroscopic guidance, in 23 patients. and it is different to further analyze the relationship between hemodynamic Ees/Ea and echocardiographic RVPAC. Then, a mix of therapies dose showed in the study. Finally, long-term follow-up was not conducted, however, large-scale, long-term, multicenter prospective studies are needed or confirmation.

## Conclusion

In conclusion, ambrisentan plus PDE-5i combination therapy resulted in a significant improvement in RV systolic function and RVPAC in patients with severe PAH. These findings provide insight into the hemodynamic and RV effects of ambrisentan plus PDE-5i combination therapy. More importantly, the RVPAC correlated with known prognostic markers of PAH. RVPAC may be an accurate non-invasive and sensitive marker of RV function and clinical improvement in response to combination therapy. Considering multiple factors such as a small number of patients, the conclusion is guarded. Therefore, future prospective studies are required to determine whether or how long combination therapy optimizes RV reverse remodeling and to evaluate treatment outcomes based on a long-term follow-up patients of severe PAH to provide further insights into clinical practice.

## Data Availability Statement

The original contributions presented in the study are included in the article/supplementary material, further inquiries can be directed to the corresponding author/s.

## Ethics Statement

The studies involving human participants were reviewed and approved by the Ethics Committee of First Affiliated Hospital of Guangxi Medical University. The patients/participants provided their written informed consent to participate in this study. Written informed consent was obtained from the individual(s) for the publication of any potentially identifiable images or data included in this article.

## Author Contributions

W-FL: conception, methodology, data curation, formal analysis, writing-original draft, and writing-review and editing. YD: conceptualization, methodology, design, and writing-review and editing. BW, KH, PD, S-SX, and D-DW: data curation. All authors have read and approved the final manuscript.

## Conflict of Interest

The authors declare that the research was conducted in the absence of any commercial or financial relationships that could be construed as a potential conflict of interest.

## Publisher’s Note

All claims expressed in this article are solely those of the authors and do not necessarily represent those of their affiliated organizations, or those of the publisher, the editors and the reviewers. Any product that may be evaluated in this article, or claim that may be made by its manufacturer, is not guaranteed or endorsed by the publisher.
